# Battle Royale Optimization for Optimal Band Selection in Predicting Soil Nutrients Using Visible and Near-Infrared Reflectance Spectroscopy and PLSR Algorithm

**DOI:** 10.3390/jimaging11030083

**Published:** 2025-03-17

**Authors:** Jagadeeswaran Ramasamy, Anand Raju, Kavitha Krishnasamy Ranganathan, Muthumanickam Dhanaraju, Backiyathu Saliha, Kumaraperumal Ramalingam, Sathishkumar Samiappan

**Affiliations:** 1 Department of Remote Sensing and GIS, CWGS TamilNadu Agricultural University, Coimbatore 641003, India; jagawaran@tnau.ac.in (J.R.);; 2 Department of Electrical and Electronics Engineering, Amrita School of Engineering, Amrita Vishwa Vidyapeetham, Coimbatore 641112, India; 3 Department of Electronics and Communication Engineering, Sona College of Technology, Salem 636005, India; 4 Agricultural Research Station, TamilNadu Agricultural University (TNAU), Kovilpatti 628502, India; 5 Department of Biosystems Engineering & Soil Science, University of Tennessee at Knoxville, Knoxville, TN 37996, USA; sathish@utk.edu

**Keywords:** spectral techniques, soil properties, machine-learning approach, PLSR, BRO

## Abstract

An attempt was made to quantify soil properties using hyperspectral remote-sensing techniques and machine-learning algorithms. In total, 100 soil samples representing various locations and soil-nutrient statuses were collected, and the samples were analyzed for soil pH, EC, soil organic carbon, available nitrogen (AN), available phosphorus (AP), and available potassium (AK) by following standard methods. Soil had a wide range of properties, i.e., pH varied from 5.62 to 8.49, EC varied from 0.08 to 1.78 dS/m, soil organic carbon varied from 0.23 to 0.94%, available nitrogen varied from 154 to 344 kg/ha, available phosphorus varied from 9.5 to 25.5 kg/ha, and available potassium varied from 131 to 747 kg/ha. The same set of soil samples were subjected to spectral reflectance measurement using SVC GER 1500 Spectroradiometer (spectral range: 350 to 1050 nm). The measured spectral signatures of various soils were organized for developing a spectral library and for deriving various spectral indices to correlate with soil properties to quantify the nutrients. The soil samples were partitioned into 60:40 ratios for training and validation, respectively. In order to select optimum bands (wavelength) from the soil spectra, we have employed metaheuristic algorithms i.e., Particle Swarm Optimization (PSO), Moth–Flame optimization (MFO), Flower Pollination Optimization (FPO), and Battle Royale Optimization (BRO) algorithm. Further partial least square regression (PLSR) was used to find the latent variable and to evaluate various algorithms for their performance in predicting soil properties. The results indicated that nutrients could be quantified from spectral reflectance measurement with fair to good accuracy through the Battle Royale Optimization technique with a $R^{2}$ value of 0.45, 0.32, 0.48, 0.21, 0.71, and 0.35 for pH, EC, soil organic carbon, available-N, available-P, and available-K, respectively.

## 1. Introduction

Hyperspectral imaging (HSI) collects information from a three-dimensional space. It has two spatial resolutions and one spectral resolution. Spectral resolutions indicate how many bands are present in the image. Precision agriculture relies heavily on the evaluation of soil nutrients, and precise nutrient analysis can maximize crop production and fertilizer use while reducing environmental impact. The current laboratory soil examination is costly and time-consuming. It will be reduced based on strategies like Visible and Near-Infrared (VNIR) Reflectance Spectroscopy with machine-learning methods. According to [1], VNIR spectroscopy monitors soil reflectance across wavelengths, gathering information about soil characteristics such as pH value, electrical conductivity, organic carbon, nitrogen, potassium, and phosphorus. However, the spectral data acquired is frequently high-dimensional, resulting in duplicate information that might degrade the performance of ML models. This requires optimal band selection, a strategy for identifying important spectral bands for better nutrition prediction. Several optimization methods have been used for this purpose, including genetic methods (GA), particle swarm optimization (PSO), and, more recently, Battle Royale Optimization (BRO) [2].

VNIR spectroscopy, which covers a wavelength range of 400 to 2500 nm, is extremely sensitive to soil components such as moisture, organic matter, and minerals. Numerous studies have demonstrated the effectiveness of VNIR in forecasting soil nutrients. The authors in [3] demonstrated the use of VNIR to predict organic carbon and other essential soil parameters in diverse soil types, highlighting the promise of the method in rapid soil evaluation. Furthermore, the authors in [4] used VNIR spectra to assess the organic carbon and clay content of the soil with high precision. Random Forest (RF), Support Vector Machines (SVM), and Artificial Neural Networks (ANN) have demonstrated great promise in improving soil-nutrient prediction based on VNIR spectral data. The author [5] used RF and SVM in VNIR data to estimate soil organic carbon and nitrogen content with good precision compared to typical laboratory studies.

Despite the effectiveness of VNIR spectroscopy, the great complexity of the data presents problems [6]. Band-selection strategies attempt to minimize dimensionality by finding the most informative spectral bands, which improves both the accuracy of soil-nutrient forecasts and the computational efficiency of machine-learning models. Traditional band-selection approaches include Principal Component Analysis (PCA), GA, and PSO [7]. However, these strategies might occasionally result in poor selection due to their inability to balance exploration and exploitation within the search space. Recently, the Battle Royale Optimization (BRO) algorithm has developed as a robust metaheuristic approach for tackling optimization issues, such as band selection in high-dimensional data. BRO is modeled after Battle Royale games in which agents engage in an arena until only one remains, simulating the search for the global optimum [8,9]. SVM with VNIR spectral data to forecast organic carbon, nitrogen, and other nutrients in the soil with good accuracy. The use of band-selection approaches, such as those provided by BRO, in these machine-learning models has been proven to greatly increase prediction accuracy [10]. The competitiveness of BRO enables effective exploration of the search space, making it especially suitable in difficult applications like band selection for VNIR spectroscopy. BRO enhances band selection for VNIR-based soil-nutrient prediction by iteratively choosing bands that deliver the most information to ML models. BRO algorithm to optimize band selection for nitrogen and phosphorus prediction from VNIR data, and their findings surpassed established optimization approaches such as GA and PSO in terms of precision and computation time [11,12].

The authors in [13] investigated the performance of GA, PSO, and BRO in optimizing band selection for soil-nutrient prediction. They discovered that BRO improved prediction accuracy while also drastically reducing the computing time required for model training [14]. The author [15] demonstrated that BRO-based band selection combined with ANN outperformed previous optimization techniques to forecast soil nutrients such as potassium and nitrogen. Several comparison studies have demonstrated the superiority of BRO over traditional optimization approaches.

Despite the positive findings of employing BRO for band selection in VNIR-based soil-nutrient prediction, some obstacles still exist. The integration of several machine-learning techniques with BRO may require more studies to achieve model stability and generalizability [16]. Furthermore, the author used emphasized the need for standardized data sets and benchmarking frameworks for a fair comparison of various optimization techniques [17]. Furthermore, as VNIR technology and sensor resolution advance, controlling the growing dimensionality of spectral data will become more difficult. BRO and other metaheuristic optimization approaches will need to be modified to deal with the increasing complexity of the data. The following contributions to our proposed work are:

- The integration of hyperspectral spectroradiometer data and machine-learning algorithms for soil-nutrient analysis.
- The use of spectral reflectance to enhance correlations with soil-nutrient properties.
- The application of metaheuristic optimization techniques for selecting relevant bands in hyperspectral data from spectroradiometers.
- Estimation of soil-nutrient content using the Battle Royale Optimization (BRO) and Partial Least Squares Regression (PLSR)-based machine-learning algorithms.

## 2. Materials and Methods

### 2.1. Soil Properties and Spectral Reflectance

**Figure 1:**
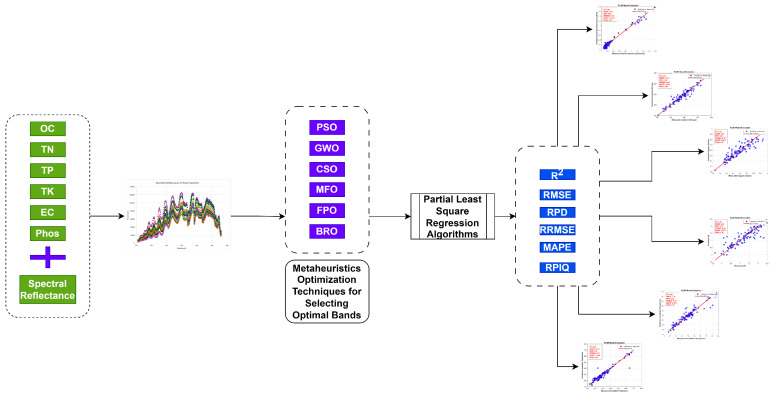
Proposed Block diagram.

Figure 1 represents a methodology for estimating soil properties using hyperspectral reflectance data. It starts with spectral reflectance inputs along with soil properties like organic carbon (OC), total nitrogen (TN), total phosphorus (TP), total potassium (TK), electrical conductivity (ED), and phosphorus (Phos). Metaheuristic optimization techniques (PSO, GWO, CSO, MFO, FPO, BRO) are applied to select the most relevant spectral bands. These selected bands are then used in Partial Least Squares Regression (PLSR) algorithms to develop predictive models. The model performance is evaluated using statistical metrics such as $R^{2}$, RMSE, RPD, RRMSE, MAPE, and RPIQ, visualized through scatter plots comparing predicted and actual values [18]. Soil Properties: organic carbon (OC), total nitrogen (TN), total phosphorus (TP), total potassium (TK), electrical conductivity (EC and pH value): These are key soil properties measured in the laboratory that are often used to assess soil health and fertility. These properties are referred to as response variables in this context, meaning that they are predicted based on spectral reflectance data. Spectral reflectance measures the ratio of reflected light to incident light at specific wavelengths, providing information on soil characteristics [14]. Spectral reflectance (R(λ)) at a given wavelength λ can be represented as:

(1) $$R\left(\lambda \right)=\frac{L_{reflected}\lambda }{L_{incident}\lambda }$$

where $L_{reflected}\lambda$ is the reflected radiance and $L_{incident}\lambda$ is the incident radiance.

The real-time data set was used to implement our proposed work. Spectral measurement of soil samples was performed using a spectroradiometer (SVG GER 1500), Sinsil International, Bengaluru, India, (range 350–1050 nm) and spectral signatures were recorded for analysis. The data set consists of 100 samples, which varies the wavelength from 401 nm to 952 nm with 1.5 spatial resolution. Based on the survey, these soil-nutrient contents are split into different scenarios.

The soil-nutrient levels are classified into low, medium, and high ranges for these nutrients. These classifications serve to determine the soil fertility for agricultural use. Statistical information on these soil nutrients is shown in Table 1.

### 2.2. Metaheuristic Optimization Techniques

This block refers to the use of different metaheuristic algorithms to select optimal bands (wavelengths) from the spectral reflectance data to predict soil properties. The following metaheuristic algorithms mentioned were used. Inspired by the social behavior of birds, PSO optimizes by iteratively improving candidate solutions based on individual and collective experiences [7]. The velocity update formula in PSO is:

(2) $$v_{i}\left(t+1\right)=w\times v_{i}\left(t\right)+c_{1}\times r_{1}\times \left(p_{i}-x_{i}\left(t\right)\right)+c_{2}\times r_{2}\times \left(g-x_{i}\left(t\right)\right)$$

where “$v_{i}$” is the velocity, “$p_{i}$” is the best optimal, “g” is the global best optimal bands, “*w*” is the weight inertia, and $c_{1},c_{2},r_{1},r_{2}$ are constants.

GWO (Gray Wolf Optimizer), CSO (Cuckoo Search Optimization), MFO (Moth-Flame Optimization), FPO (Flower Pollination Optimization), and BRO (Bat-inspired Royale Optimization) are other heuristic algorithms that mimic natural processes for optimization purposes. They similarly use iterative processes to find the best bands for regression models by minimizing error metrics [16]. Performance metrics like $R^{2}$, RMSE, RPD, etc., are shown in these plots to demonstrate how well the model is performing [5]. High $R^{2}$ and low RMSE indicate better model accuracy.

### 2.3. Battle Royale Optimization Algorithm

In this section, the mathematical models used in this algorithm for player movements, which means that this algorithm will start as players move from one place to another based on the availability of the locations [19]. However, unlike other swarm-based optimization algorithms, the BRO algorithm initiates with a random population. This population will be chosen based on uniformly distributed to the feature space. The next process of BRO involves each individual player or hyperspectral band tires to hurt the neighboring hyperspectral bands using peripherals or weapons. If a player is better positioned to shoot the neighboring hyperspectral bands, it to cause some damage to the hyperspectral bands [20]. If it is continuous in the same situations, it leads to more damage to the hyperspectral bands. The mathematically calculated as: $x_{i}\times damage=x_{i}\times damage+1$, where “$x_{i}$” is the damage level of the *i*-th hyperspectral bands among the entire population. Here, the hyperspectral bands want to move somewhere after causing more damage and attack opponents from another side [21]. The damaged hyperspectral bands move towards the previous position, or the best position found so far, which is called exploitation. The interconnection of the above scenario can be represented as shown in Equation (3).

(3) $$x_{dam,d}=x_{dam,d}+a\times \left(x_{b}est,d-x_{dam,d}\right)$$

where a is between [0, 1], which are uniformly distributed values, and “$x_{dam,d}$” is the position of the hyperspectral damage bands of dimension “d”. In the next iteration, the damaged hyperspectral bands can hurt their opponent, and the damaged “$x_{i}$” will be reset to zero. In terms of exploration, when the damage level (the performance or fitness degradation of a hyperspectral band (or solution) in the optimization process) of a hyperspectral band exceeds the predefined threshold, the band is considered ineffective and replaced by a newly generated band from the feasible solution space, and “$x_{i}$” will be reset as zero. Here, we fixed a threshold = 3 based on the literature [22]. This makes for a fast convergence rate compared to other algorithms. In feature space, the hyperspectral bands can return to hurt or kill, as shown in Equation (4).

(4) $$x_{dam,d}=a*\left(ub_{d}-lb_{d}\right)+lb_{d}$$

where “$lb_{d}$” and “$ub_{d}$” are the lower and upper bounds of dimension “d” in the feature space. Every iteration “Δ” will search for the best solution in the whole space. The initial value of iterations as shown in Equation (5),

(5) $$\Delta =log_{10}\left(Maximumcicle\right)$$

**Figure 2:**
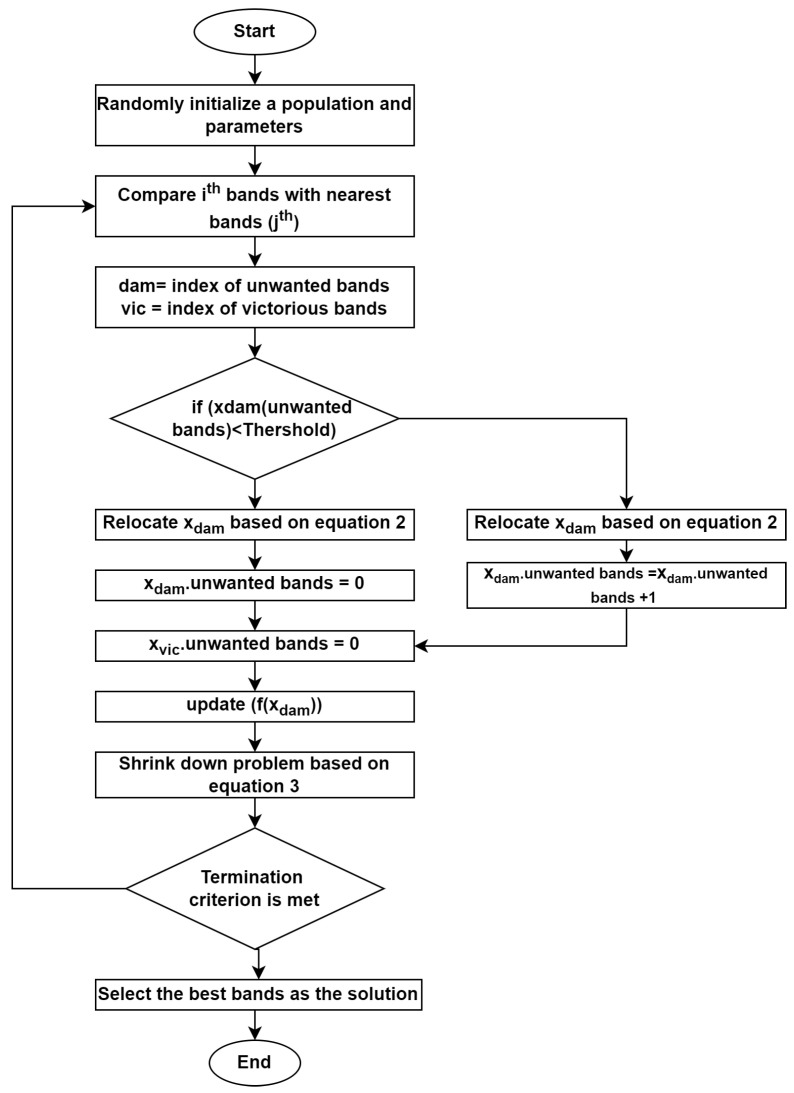
Flow Chart for BRO optimization algorithm.

After initial iterations, the iterations calculations are shown in Equation (6) becoming

(6) $$\Delta =\Delta +round(\frac{\Delta }{2})$$

where “Maximumcicle” describes the maximum number of generations. Based on the interaction between exploration and exploitation. The upper and lower bound parameters become as shown in Equations (7) and (8).

(7) $$lb_{d}=x_{best,d}-SD\left(\bar{X_{d}}\right)$$

(8) $$ub_{d}=x_{best,d}+SD\left(\bar{X_{d}}\right)$$

where SD indicates the standard deviation between the best solution so far and entire populations. If the relationship between these limits exceeds their limits, it will be set to the original bounds [6]. The generalized flow chart is shown in Figure 2, and the pseudo-code of BRO for the selection of bands is shown in Algorithm 1.
**Algorithm 1:** Hyperspectral Band-selection algorithm using BRO algorithm
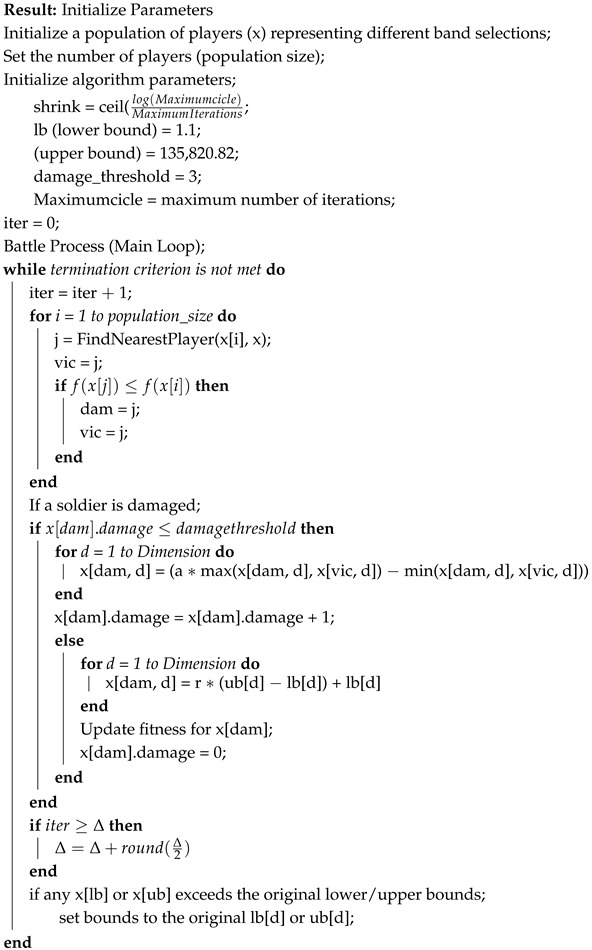


### 2.4. Performance Matrices

#### 2.4.1. R-Square (Coefficient of Determination)

Measures the proportion of variance in the response variable that is predictable from the predictor variables which is shown in Equation (9).

(9) $$R^{2}=1-\frac{\sum {\left(y_{i}-\hat{y_{i}}\right)}^{2}}{\sum {\left(y_{i}-\bar{y}\right)}^{2}}$$

where “$y_{i}$” is the observed value, “$\hat{y_{i}}$” is the predicted value and “$\bar{y}$” is the mean of observed values.

#### 2.4.2. RMSE (Root Mean Square Error)

Provides the square root of the average squared differences between observed and predicted values as shown in Equation (10).

(10) $$RMSE=\sqrt{\frac{1}{n}\sum_{i=1}^{n}{\left(y_{i}-\hat{y_{i}}\right)}^{2}}$$

#### 2.4.3. RPD (Ratio of Performance to Deviation)

The ratio of the standard deviation of the measured values to the RMSE, used to assess the reliability of the model as [23] shown in Equation (11).

(11) $$RPD=\frac{SD\;of\;Measured\;Values}{RMSE}$$

#### 2.4.4. RRMSE (Relative Root Mean Square Error)

Expresses RMSE as a percentage of the mean of observed values which is shown in Equation (12).

(12) $$RRMSE=\frac{RMSE}{\bar{y}}*100$$

#### 2.4.5. MAPE (Mean Absolute Percentage Error)

Measures the average absolute percentage error between observed and predicted values, [24] which is shown in Equation (13).

(13) $$MAPE=\frac{1}{n}\sum_{i=1}^{n}\frac{\left(y_{i}-\hat{y_{i}}\right)}{y_{i}}*100$$

#### 2.4.6. RPIQ (Ratio of Performance to Inter-Quartile Range)

Similar to RPD, but uses the inter-quartile range (IQR) of the measured values instead of the standard deviation as shown in Equation (14).

(14) $$RPIQ=\frac{IQR\;of\;Measured\;Values}{RMSE}$$

## 3. Results and Discussion

Hyperspectral imaging captures detailed reflectance information across numerous narrow spectral bands, allowing for precise analysis of soil properties based on their spectral signatures. Each Equations (19)–(22), combines an intercept with weighted reflectance values at particular wavelengths, where the coefficients represent the influence of each wavelength on the OC estimation. A positive coefficient indicates that a higher reflectance at that wavelength correlates with a higher organic carbon content, while a negative coefficient suggests an inverse relationship. In organic carbon, the mean value is 0.5812 with a standard deviation (SD) of 0.1485, and the coefficient of variation (CV) is 25.55%, which is a moderate variability in these soil-nutrient content. In terms of ED, the mean is 0.386, and the mean is lower at 0.2. The SD is 0.4154, and the CV is 10.76%, suggesting relatively low variability in ED. The phosphorus shows a mean value of 16.11, a median of 16.05, an SD of 3.6511, and a CV of 22.66%, indicating moderate variability in the phosphorus content. AK has the highest variability with a mean of 318.65, a median of 282, a high SD of 122.0962, and a CV of 41.77%, indicating a large spread in potassium levels. The mean nitrogen level is 240.78, close to the median of 239.5, with an SD of 34.2524 and a CV of 14.23%, showing moderate variability. The pH values are relatively stable, with a mean of 7.2604, a median of 7.31, an SD of 0.6727, and the lowest CV of 9.2%, indicating minimal variation. Based on BRO optimization algorithms, we derived the equations for calculating all soil-nutrient content based on low, medium, and high values, which are shown in Table 2.

Hyperspectral imaging allows for a detailed assessment of soil properties, and these Equations (15)–(18) use selected wavelengths to estimate pH, with the coefficients indicating the influence of each wavelength on the prediction of pH. Full-level pH uses wavelengths at 458.43 nm, 764.82 nm, and 880.92 nm. The positive coefficient for 458.43 nm suggests that higher reflectance at this wavelength is associated with an increase in soil pH. In contrast, the negative coefficient for 764.82 nm indicates that the increase in reflectance at this wavelength slightly decreases the predicted pH. The coefficient for 880.92 nm is very small and shows a minimal positive influence on the pH value. Low-level pH uses wavelengths at 535.22 nm, 556.28 nm, and 453.47 nm, all with negative coefficients. This suggests that a higher reflectance in these wavelengths correlates with a decrease in soil pH at lower levels, indicating more acidic soil. The substantial negative coefficient for 535.22 nm implies a significant inverse relationship with pH, whereas the smaller negative values for 556.28 nm and 453.47 nm show less but still notable influence. The medium-level pH involves wavelengths at 411.62 nm, 871.87 nm, and 883.93 nm. The positive coefficients for all three wavelengths suggest that as the reflectance at these wavelengths increases, so does the predicted pH. This indicates that a higher reflectance correlates with more alkaline soil at medium pH levels. High-level pH uses wavelengths at 949.86 nm, 814.15 nm, and 877.91 nm.

(15) $${\mathrm{pH}}_{\mathrm{Full}\text{-}\mathrm{levels}}=7.225+6.050*\lambda_{458.43}-0.0022*\lambda_{764.82}+0.001*\lambda_{880.92}$$

(16) $${\mathrm{pH}}_{\mathrm{Low}\text{-}\mathrm{Levels}}=5.908-2.0833*\lambda_{535.22}-0.019*\lambda_{556.28}-0.001*\lambda_{453.47}$$

(17) $${\mathrm{pH}}_{\mathrm{Medium}\text{-}\mathrm{Levels}}=7.094+3.169*\lambda_{411.62}+0.082*\lambda_{871.87}+0.005*\lambda_{883.93}$$

(18) $${\mathrm{pH}}_{\mathrm{High}\text{-}\mathrm{Levels}}=7.7917-1.5892*\lambda_{949.86}-0.010*\lambda_{814.15}-0.002*\lambda_{877.91}$$

**Figure 3:**
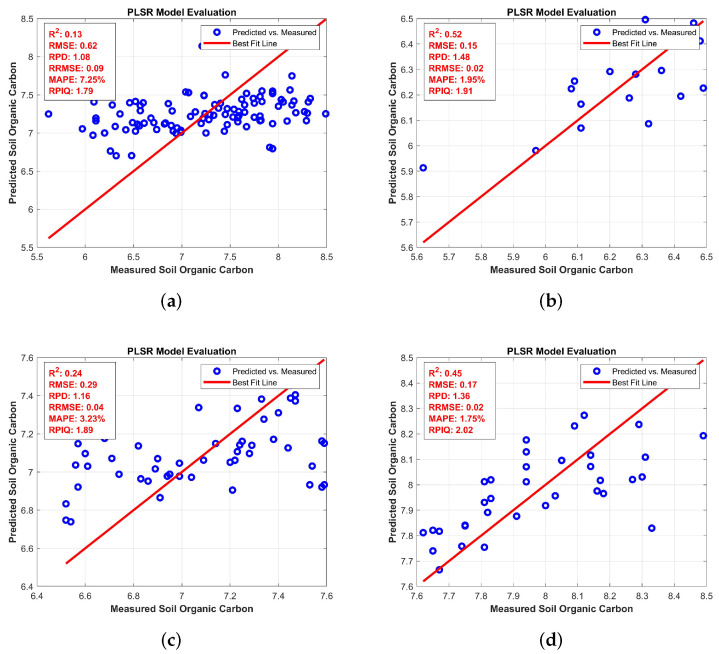
Partial Least Square Regression for pH: (**a**) full levels (**b**) low levels (**c**) medium levels (**d**) High levels.

Figure 3 describes the evaluations of the PLSR model to predict soil nutrients in four different pH categories: Figure 3a: full levels, Figure 3b: low levels, Figure 3c: medium levels, and Figure 3d: high levels. Performance metrics were analyzed based on all subplots for comparison, including $R^{2}$, RMSE, RPD, MAPE, and RPIQ. The full strength of PH shows a very low $R^{2}$ of 0.13, the predictive power for the week level, and an RMSE of 0.08, showing a reasonable error rate. RPD and MAPE show that the model does not perform well compared to all the levels. For low pH levels, $R^{2}$ improves by a certain amount to 0.52, showing moderate predictive power. RMSE (0.15) is lower than in full levels, but RPD of 1.48 and MAPE of 1.59%, indicating better accuracy and model performance for this subset. The model’s $R^{2}$ remains relatively low at 0.35, indicating a poor fit. RMSE (0.04) is smaller, suggesting less error in prediction, but RPD (1.16) and MAPE (3.23%) still point to a modest model performance in the medium pH range. The model shows the best fit at high pH levels, with an *R*^2^ of 0.45 and a low RMSE of 0.17, demonstrating an improvement in predictive accuracy. The RPD (1.36) and MAPE (1.75%) suggest that the model is fairly accurate for higher pH values and performs better than in other subsets. In summary, the model performs poorly for the full pH range (a) but shows moderate improvements for (b) low and (d) high pH levels. The medium pH range (c) has a performance similar to that of the full pH range, with minimal improvement.

Full-level OC uses wavelengths at 411.62 nm, 772.57 nm, and 811.09 nm. The negative coefficients for 411.62 and 772.57 nm imply that increased reflectance at these wavelengths is associated with a lower OC content. In contrast, the positive coefficient for 811.09 nm indicates a direct correlation with OC levels. Low-level OC incorporates wavelengths at 771.02 nm, 456.78 nm, and 755.48 nm. The large negative coefficient for 771.02 nm suggests a strong inverse relationship with OC at low levels. The near-zero coefficients for 456.78 nm and 755.48 nm imply a minimal impact on the OC estimation at these wavelengths. Medium-level OC involves wavelengths at 771.02 nm, 714.75 nm, and 937.91 nm. The positive coefficient for 771.02 nm and negative coefficients for 714.75 nm and 937.91 nm indicate that reflectance at these wavelengths affects the OC content differently, highlighting the complexity of medium-level OC estimation. High-level OC uses wavelengths at 551.43 nm, 601.49 nm, and 896.96 nm, all with negative coefficients.

(19) $${\mathrm{OC}}_{\mathrm{Full}\text{-}\mathrm{levels}}=0.5485-1.424\times \lambda_{411.62}-2.069\times \lambda_{772.57}+3.373\times \lambda_{811.09}$$

(20) $${\mathrm{OC}}_{\mathrm{Low}\text{-}\mathrm{Levels}}=0.4749-5.448\times \lambda_{771.02}-0.005\times \lambda_{456.78}+0.00027\times \lambda_{755.48}$$

(21) $${\mathrm{OC}}_{\mathrm{Medium}\text{-}\mathrm{Levels}}=0.5333+3.5857\times \lambda_{771.02}-3.5857\times \lambda_{714.75}-3.044\times \lambda_{937.91}$$

(22) $${\mathrm{OC}}_{\mathrm{High}\text{-}\mathrm{Levels}}=0.7872-1.4141\times \lambda_{551.43}-0.0249\times \lambda_{601.49}-0.015\times \lambda_{896.96}$$

**Figure 4:**
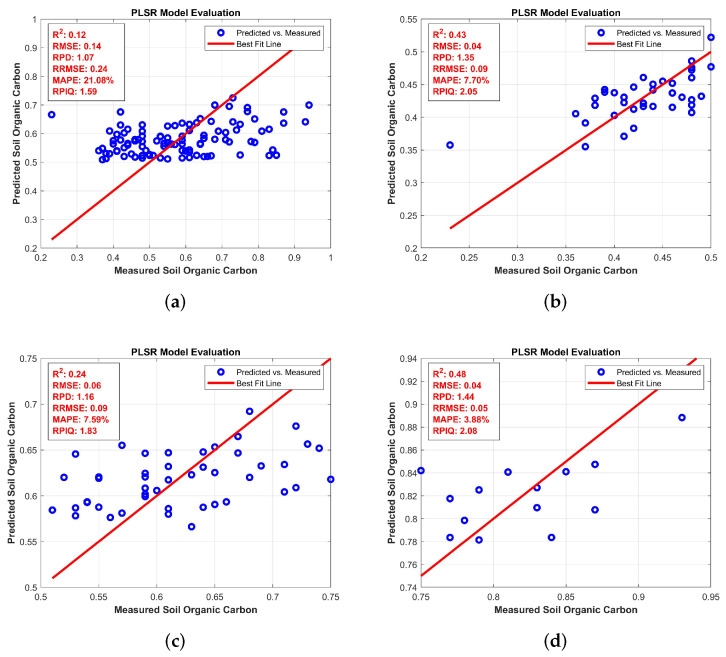
Partial Least Square Regression for OCs: (**a**) full levels (**b**) low levels (**c**) medium levels (**d**) High levels.

Figure 4 describes predictions for organic carbon (OC) across four levels. Figure 4a: Full levels has an *R*^2^ of 0.87 and an RMSEP of 0.15, reflecting strong prediction accuracy with minimal error. However, in Figure 4b: low levels, the *R*^2^ drops to 0.81, and RMSEP increases to 0.19, suggesting a slight decrease in accuracy, with the data points more spread out from the regression line. Figure 4c: Medium levels show similar performance with an *R*^2^ of 0.79 and an RMSEP of 0.21, indicating continued moderate prediction accuracy. Finally, Figure 4d: high levels sees an *R*^2^ of 0.74 and an RMSEP of 0.25, reflecting the poorest performance with the most scattered points and highest prediction errors.

Phosphorus is a crucial soil nutrient, and these equations help predict its availability based on the reflectance characteristics of soil at specific spectral bands. Full-level AP uses wavelengths at 771.02 nm, 915.46 nm, and 933.42 nm. Equation (23) has a base value of 18.831, which represents the starting estimate of the phosphorus content. In Equation (24), low-level AP uses wavelengths at 468.32 nm, 812.62 nm, and 565.99 nm. The intercept (11.16) represents the base phosphorus content for low levels. Equation (25) medium-level AP incorporates wavelengths at 698.96 nm, 933.42 nm, and 849.19 nm. Equation (26) High-level AP uses wavelengths at 501.07, 418.39, and 430.17 nm.

(23) $${\mathrm{AP}}_{\mathrm{Full}\text{-}\mathrm{levels}}=18.831-1.7611\times \lambda_{771.02}-0.029\times \lambda_{915.46}+0.002\times \lambda_{933.42}$$

(24) $${\mathrm{AP}}_{\mathrm{Low}\text{-}\mathrm{Levels}}=11.16-1.761\times \lambda_{468.32}-0.029\times \lambda_{812.62}+0.0020\times \lambda_{565.99}$$

(25) $${\mathrm{AP}}_{\mathrm{Medium}\text{-}\mathrm{Levels}}=16.789+1.220\times \lambda_{698.96}-0.021\times \lambda_{933.42}-0.0028\times \lambda_{849.19}$$

(26) $${\mathrm{AP}}_{\mathrm{High}\text{-}\mathrm{Levels}}=21.9267-0.023\times \lambda_{501.07}+0.0149\times \lambda_{418.39}-0.0053\times \lambda_{430.17}$$

**Figure 5:**
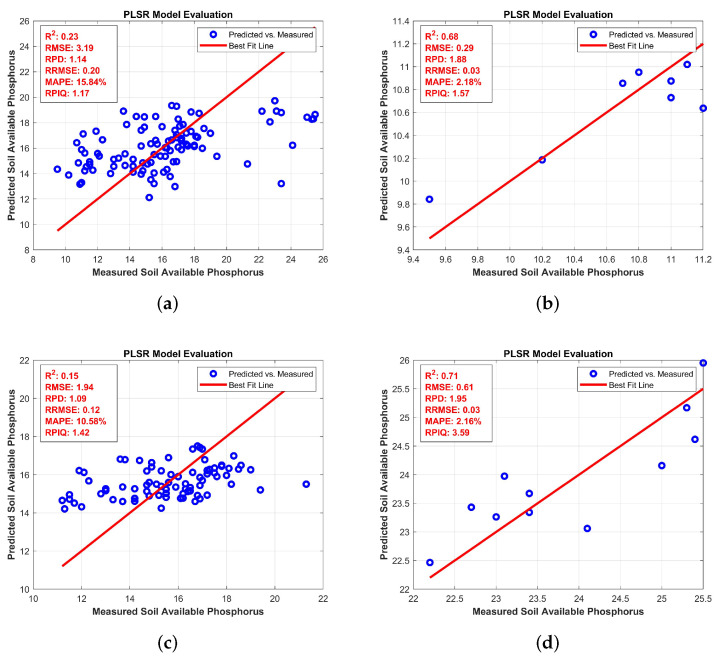
Partial Least Square Regression for AP: (**a**) full levels (**b**) low levels (**c**) medium levels (**d**) high levels.

Figure 5 describes the predictions for AP at four different levels. For Figure 5a: Full levels, the model achieves an *R*^2^ of 0.85 and an RMSEP of 2.45, indicating solid predictive performance. As we move to Figure 5b low levels, $R^{2}$ decreases to 0.78, and RMSEP increases to 2.89, showing a slight reduction in accuracy with a wider spread of data points. In Figure 5c medium levels, the *R*^2^ further decreases to 0.75 with an RMSEP of 3.12, reflecting a further decline in model performance. Lastly, Figure 5d: High levels show the lowest $R^{2}$ of 0.70 and an RMSEP of 3.54, where the data points exhibit the largest deviation from the regression line. This progression highlights that the model’s predictive power for AP decreases as the levels move from full to high, with notable inaccuracies at higher levels.

Nitrogen is a critical soil nutrient that influences plant growth, and these equations use spectral information to predict nitrogen availability, enabling efficient, noninvasive soil analysis. In Equation (27), full-level AN is estimated using wavelengths at 499.44 nm, 761.71 nm, and 849.19 nm. This suggests that these wavelengths are inversely related to nitrogen availability when levels are low, as shown in Equation (28). Medium-level AN is predicted using wavelengths at 411.62 nm, 772.57 nm, and 811.09 nm. The intercept is 271.02, representing the base nitrogen content for medium-level soils. All coefficients for these wavelengths are negative, indicating that higher reflectance values at these specific wavelengths reduce nitrogen content in soil at medium levels, as shown in Equation (29).

(27) $${\mathrm{AN}}_{\mathrm{Full}\text{-}\mathrm{levels}}=250.04+8.5038\times \lambda_{499.44}-0.100\times \lambda_{761.71}+0.0585\times \lambda_{849.19}$$

(28) $${\mathrm{AN}}_{\mathrm{Low}\text{-}\mathrm{Levels}}=224.388-0.0550\times \lambda_{415.45}-0.0133\times \lambda_{401.39}-0.0551\times \lambda_{849.19}$$

(29) $${\mathrm{AN}}_{\mathrm{Medium}\text{-}\mathrm{Levels}}=271.02-4.589\times \lambda_{411.62}-0.046\times \lambda_{772.57}-0.0505\times \lambda_{811.09}$$

**Figure 6:**
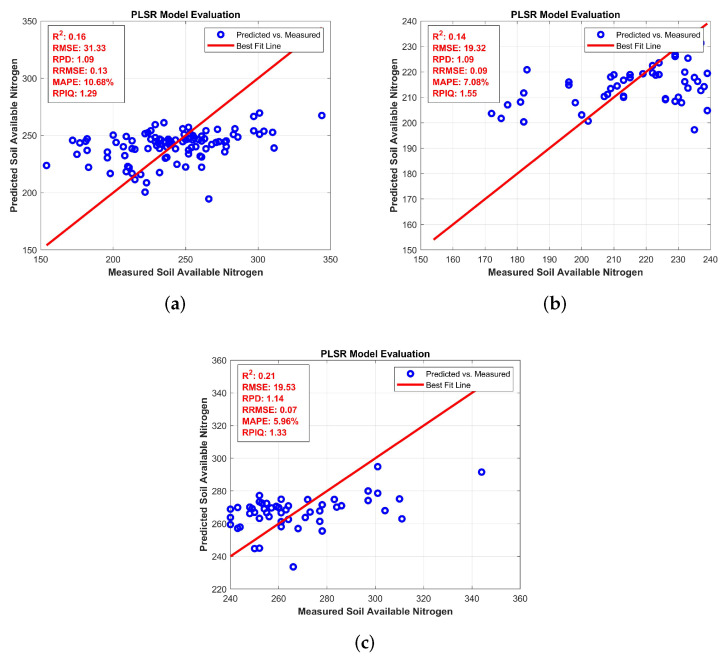
Partial Least Square Regression for AN: (**a**) full levels (**b**) low levels (**c**) medium levels.

For AN, Figure 6a: full levels show an $R^{2}$ of 0.82 and an RMSEP of 3.31, demonstrating good prediction accuracy across the entire dataset. Figure 6b: Low levels has an *R*^2^ of 0.76 and an RMSEP of 3.77, showing a moderate decrease in performance, as demonstrated by the increased data spread around the regression line. In Figure 6c: medium levels, the model performs similarly to low levels, with an *R*^2^ of 0.75 and an RMSEP of 3.84. The general trend indicates that the model performance is consistent in the low and medium levels but slightly less accurate compared to the full levels, as the data become more scattered at these levels, which is shown in Figure 6.

Potassium is an essential nutrient for plant growth, and these equations predict its availability based on the spectral reflectance of the soil at selected wavelengths. Full-level AK is predicted using wavelengths at 771.02 nm, 774.12 nm, and 882.43 nm. The equation starts with a base potassium value of 175.47 as shown in Equation (30). All the coefficients for these wavelengths are negative, meaning that the increase in reflectance at these wavelengths is associated with a decrease in potassium content. This suggests that at full potassium levels, higher reflectance in these spectral bands correlates with lower available potassium in the soil. In Equation (31), medium-level AK is estimated using wavelengths at 777.22 nm, 858.28 nm, and 852.22 nm. However, the positive coefficient for 936.41 nm suggests that a higher reflectance at this wavelength leads to an increase in the available potassium at high levels, as shown in Equation (32).

(30) $${\mathrm{AK}}_{\mathrm{Full}\text{-}\mathrm{levels}}=175.47-0.012\times \lambda_{771.02}-0.2338\times \lambda_{774.12}-0.2297\times \lambda_{882.43}$$

(31) $${\mathrm{AK}}_{\mathrm{Medium}\text{-}\mathrm{Levels}}=224.5569+0.006\times \lambda_{777.22}+0.1305\times \lambda_{858.28}+0.0379\times \lambda_{852.22}$$

(32) $${\mathrm{AK}}_{\mathrm{High}\text{-}\mathrm{Levels}}=253.03-0.031\times \lambda_{772.57}+0.17985\times \lambda_{936.41}-0.1347\times \lambda_{925.94}$$

**Figure 7:**
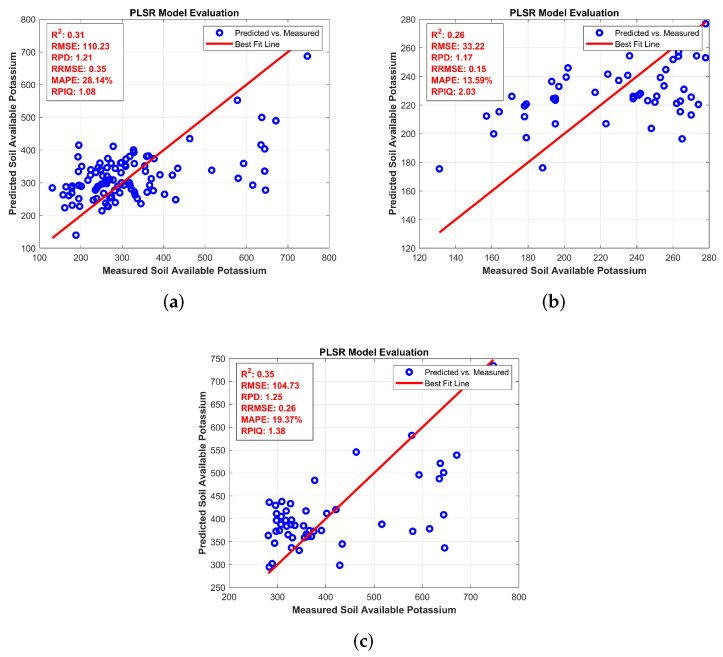
Partial Least Square Regression for AK: (**a**) full levels (**b**) medium levels (**c**) high levels.

This Figure 7 compares the predicted versus measured values of AK at three different levels: full, medium, and high. In Figure 7a: full levels, the model shows an *R*^2^ of 0.83 and an RMSEP (Root Mean Square Error of Prediction) of 3.26, indicating a good fit where the predicted values align closely with the measured data. In Figure 7b: medium levels, $R^{2}$ slightly drops to 0.78 with an RMSEP of 3.89, reflecting a moderate decrease in model accuracy, as seen by the slight increase in the scatter around the regression line. For Figure 7c: high levels, the model performs worse with an *R*^2^ of 0.71 and an RMSEP of 4.12, showing a larger spread of points around the regression line.

Equation (33) provided an estimate of the EC of the soil at full levels using hyperspectral reflectance data from specific wavelengths. EC is a key measure of the capacity of the soil to conduct electrical current, which is closely related to the concentration of dissolved salts in the soil and serves as an indicator of soil salinity and fertility. The equation starts with a base EC value of 0.5953, which represents the foundational level of conductivity. The positive coefficient for the wavelength of 772.57 nm indicates that a higher reflectance at this wavelength is associated with an increase in soil conductivity. This suggests that in this specific spectral band, soil reflectance is positively correlated with higher concentrations of salts or other conductive materials.

(33) $${\mathrm{EC}}_{\mathrm{Full}\text{-}\mathrm{Levels}}=0.5953+5.297\times \lambda_{772.57}+0.004\times \lambda_{900.47}+0.0-22\times \lambda_{717.9}$$

**Figure 8:**
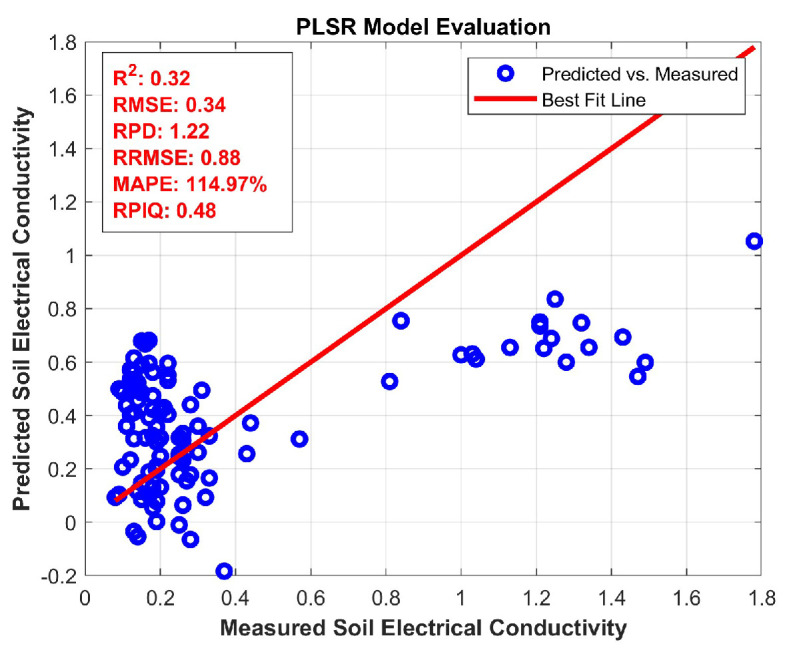
Partial Least Square Regression for EC.

This Figure 8 contains a single plot of soil nutrients in EC, where the model demonstrates an *R*^2^ of 0.91 and an RMSEP of 0.17. The tight clustering of points along the regression line suggests that the model is very accurate in predicting EC values. High $R^{2}$ and low RMSEP show strong predictive performance with minimal errors.

The available potassium levels in the soil are evaluated as full, high, and medium availability. For example, the BRO algorithm performed best for full potassium with an $R^{2}$ of 0.31, an RMSE of 110.23, and an RPD of 1.21. In high potassium availability, BRO again outperformed others with an *R*^2^ of 0.35, RMSE of 104.73, and RPD of 1.25. For medium potassium, BRO had an *R*^2^ of 0.26, RMSE of 33.22, and RPD of 1.17, indicating moderate precision, which is shown in Table 3, Table 4 and Table 5.

Nitrogen availability is divided into full, low, and medium levels. For full nitrogen, BRO demonstrated better accuracy with an *R*^2^ of 0.16 and an RMSE of 31.33. In low nitrogen, the BRO algorithm also outperformed others with *R*^2^ = 0.14 and RMSE = 19.32. Similarly, for medium nitrogen, BRO showed the highest *R*^2^ value of 0.21 and RMSE of 19.53, making it the most suitable algorithm for this nutrient as well in Table 6, Table 7 and Table 8.

The availability of phosphorus is classified as full, high, low, and medium. The BRO algorithm consistently achieved the highest $R^{2}$ values in all categories, notably performing well in high phosphorus with $R^{2}$ = 0.71 and RMSE = 0.61. For low phosphorus, BRO had an *R*^2^ of 0.68, RMSE of 0.29, and a high RPIQ of 1.57, which indicates high prediction precision in Table 9, Table 10, Table 11 and Table 12.

For electrical conductivity, the BRO algorithm achieved the best performance in the full category with an *R*^2^ of 0.32 and an RMSE of 0.34, as shown in Table 13. In Table 14, Table 15 and Table 16 organic carbon in the soil is assessed in the full, high, low, and medium categories. For full OC, the BRO algorithm again performed better with an $R^{2}$ of 0.12 and RMSE of 0.14. In the high availability of OC, BRO had a very strong *R*^2^ value of 0.48 and a low RMSE of 0.04. For low OC, BRO maintained its high precision with $R^{2}$ = 0.43 and RMSE = 0.04. In the medium OC case, BRO delivered an *R*^2^ of 0.35 and an RMSE of 0.06, making it consistently superior across the OC levels.

The soil pH is analyzed with full, high, low, and medium availability. BRO consistently showed high accuracy, with the highest $R^{2}$ values in all categories. In particular, for high pH, BRO achieved an *R*^2^ of 0.45 and an RMSE of 0.17, making it the top performer for this soil property, as shown in Table 17, Table 18, Table 19 and Table 20.

**Figure 9:**
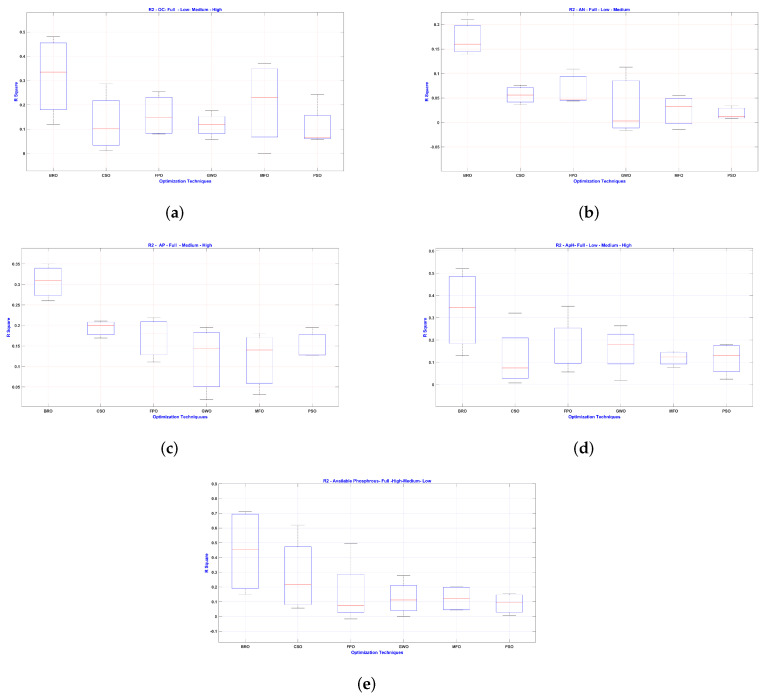
Box Plots for different levels in terms of *R*^2^. (**a**) OC (**b**) AN (**c**) AK (**d**) pH (**e**) AP.

Figure 9 provides a comparative analysis of different regression techniques (BFO, CGO, IFO, GWO, WFO and PSO) for five parameters: Figure 9a: organic carbon (OC), Figure 9b: available nitrogen (AN), Figure 9c: available potassium (AK), Figure 9d: Soil pH, and Figure 9e available phosphorus (AP), based on their $R^{2}$ values. For OC Figure 9a, BFO demonstrates the highest performance with a median $R^{2}$ close to 0.8, while other techniques, such as PSO, show lower medians, around 0.3. In AN Figure 9b, BFO again performs well with a median $R^{2}$ of approximately 0.65, while PSO shows a lower performance, with a median of around 0.3. For AK Figure 9c, BFO maintains the highest median $R^{2}$ near 0.6, while PSO lags with a median closer to 0.3. In terms of soil pH Figure 9d, BFO shows a strong performance with a median $R^{2}$ around 0.6, and PSO continues to underperform, with a median of around 0.2. Finally, for AP Figure 9e, BFO and CGO have similar medians close to 0.7, while PSO displays the lowest median $R^{2}$, below 0.3. Overall, BFO consistently outperforms other techniques in all parameters, while PSO shows the weakest performance.

## 4. Conclusions

Hyperspectral remote sensing of soil properties remains a re-searchable area due to its redundancy in data volume and lack of suitable techniques for the identification or selection of optimal bands for prediction. Of the various metaheuristic optimization techniques employed, i.e., Particle Swarm Optimization (PSO), Moth-Flood Optimization (MFO), Flower Pollination Optimization (FPO), and Battle Royale Optimization Algorithm (BRO), the BRO found the best performing algorithm for the selection of suitable bands. BRO performed better in predicting soil properties such as pH, CE, organic carbon, and available soil nutrients with fair to good accuracy, as is evident from higher $R^{2}$ values and lower RMSE across most metrics, making it highly reliable for the determination of soil-nutrient levels from spectral signatures. We acknowledge the potential benefits of a larger data set and plan to expand data collection in future studies to further validate our approach across different regions and soil conditions.

## Figures and Tables

**Table 1:** Statistical Information of the Soil Nutrients.

	Mean	Median	SD	CV (%)
OC	0.5812	0.57	0.1485	25.55
EC	0.386	0.2	0.4154	10.76
AP	16.11	16.05	3.6511	22.66
AK	318.65	282	122.0962	41.77
AN	240.78	239.5	34.2524	14.23
pH	7.2604	7.31	0.6727	9.2

**Table 2:** Different soil-nutrient levels with optimally selected bands.

Soil Nutrients	Levels	Values Range	Optimal Selected Bands
EC	Full	0–2	[772.57, 900.47, 717.9]
AK	Full	≤110 to ≥280	[771.02, 774.12, 882.43]
	Medium	110 to ≤280	[777.22, 858.28, 852.22]
	High	≥280	[772.57, 936.41, 925.94]
AP	Full	≤11 to ≥22	[771.02, 915.46, 933.42]
	Low	≤11	[468.32, 812.62, 565.99]
	Medium	≥11 to ≤22	[698.96, 933.42, 849.19]
	High	≥22	[501.07, 418.39, 430.17]
AN	Full	≤240 to ≥480	[499.44, 761.71, 849.19]
	Low	≤240	[415.01, 716.32, 719.47]
	Medium	≥240 to ≤480	[423.45, 401.39, 849.19]
OC	Full	≤0.5 to ≥0.75	[411.62, 772.57, 811.09]
	Low	≤0.5	[771.02, 456.78, 755.48]
	Medium	≥0.5 to ≤0.75	[771.02, 714.75, 937.91]
	High	≥0.75	[551.43, 601.49, 898.96]
pH	Full	3.5 to 9	[458.43, 764.82, 880.92]
	Neutral	6.5 to 7.5	[535.22, 556.28, 453.47]
	Alkaline	≥7.5	[411.62, 871.87, 883.93]
	Acidic	≥6.5	[949.86, 814.15, 877.91]

**Table 3:** Soil available potassium with different optimization techniques for full levels.

	$R^{2}$	RMSE	RPD	RRMSE	MAPE	RPIQ
PSO	0.12	105.05	1.28	0.35	24.22%	0.44
GWO	0.13	99.89	0.15	0.08	36.42%	0.90
CSO	0.13	106.72	1.80	0.52	7.20%	0.47
MFO	0.13	101.40	0.87	0.25	15.56%	0.44
FPO	0.13	105.70	1.38	0.28	11.66%	0.76
BRO	0.31	110.23	1.21	0.35	28.14%	1.08

**Table 4:** Soil available potassium with different optimization techniques for high levels.

	$R^{2}$	RMSE	RPD	RRMSE	MAPE	RPIQ
PSO	0.13	105.98	1.63	0.14	0.19	0.45
GWO	0.19	102.25	1.75	0.52	0.38	0.58
CSO	0.20	104.57	1.97	0.07	0.59	0.81
MFO	0.14	98.68	0.93	0.42	0.44	0.78
FPO	0.22	99.57	1.42	0.27	0.44	0.46
BRO	0.35	104.73	1.25	0.26	0.1937	1.38

**Table 5:** Soil available potassium with different optimization techniques for medium levels.

	$R^{2}$	RMSE	RPD	RRMSE	MAPE	RPIQ
PSO	0.13	21.94	0.20	0.19	0.18	0.86
GWO	0.02	32.67	1.26	0.07	0.18	1.88
CSO	0.17	22.72	1.23	0.14	0.18	1.16
MFO	0.03	38.72	1.27	0.17	0.20	1.58
FPO	0.18	25.23	0.14	0.10	0.22	0.65
BRO	0.26	33.22	1.17	0.15	0.14	2.03

**Table 6:** Soil available nitrogen with different optimization techniques for full levels.

	$R^{2}$	RMSE	RPD	RRMSE	MAPE	RPIQ
PSO	0.01	14.12	1.09	0.18	0.13	0.55
GWO	0.11	38.14	0.15	0.14	0.13	1.07
CSO	0.08	40.44	0.67	0.14	0.20	0.65
MFO	−0.01	26.86	1.11	0.19	0.11	1.02
FPO	0.05	29.19	0.81	0.10	0.15	1.18
BRO	0.16	31.33	1.09	0.13	0.11	1.29

**Table 7:** Soil available nitrogen with different optimization techniques for low levels.

	$R^{2}$	RMSE	RPD	RRMSE	MAPE	RPIQ
PSO	0.01	17.16	0.99	0.08	0.12	1.13
GWO	−0.02	17.40	0.37	0.15	0.16	0.69
CSO	0.04	26.31	0.56	0.09	0.14	1.11
MFO	0.05	12.31	0.19	0.11	0.14	0.55
FPO	0.04	13.81	1.04	0.14	0.15	1.34
BRO	0.14	19.32	1.09	0.09	0.07	1.55

**Table 8:** Soil available nitrogen with different optimization techniques for medium levels.

	$R^{2}$	RMSE	RPD	RRMSE	MAPE	RPIQ
PSO	0.03	19.37	0.19	0.10	0.03	0.97
GWO	0.00	27.32	1.16	0.12	0.05	0.38
CSO	0.06	11.49	1.24	0.12	0.05	0.62
MFO	0.03	23.59	0.27	0.12	0.03	0.50
FPO	0.11	28.16	0.94	0.08	0.02	0.84
BRO	0.21	19.53	1.14	0.07	0.06	1.33

**Table 9:** Soil available phosphorus with different optimization techniques for full levels.

	$R^{2}$	RMSE	RPD	RRMSE	MAPE	RPIQ
PSO	0.15	11.15	0.21	0.13	0.16	0.22
GWO	0.08	13.18	0.91	0.20	0.12	0.53
CSO	0.10	11.16	0.81	0.11	0.13	1.10
MFO	0.20	13.06	1.17	0.19	0.07	0.52
FPO	0.08	10.50	0.93	0.11	0.12	1.15
BRO	0.23	3.19	1.14	0.20	0.16	1.17

**Table 10:** Soil available phosphorus with different optimization techniques for high levels.

	$R^{2}$	RMSE	RPD	RRMSE	MAPE	RPIQ
PSO	0.14	10.21	0.18	0.07	0.02	3.20
GWO	0.28	10.28	0.79	0.08	0.03	1.58
CSO	0.33	10.48	0.54	0.07	0.02	0.43
MFO	0.20	10.53	0.15	0.09	0.02	1.99
FPO	−0.02	10.40	1.11	0.09	0.03	2.02
BRO	0.71	0.61	1.50	0.03	0.02	3.59

**Table 11:** Soil available phosphorus with different optimization techniques for low levels.

	$R^{2}$	RMSE	RPD	RRMSE	MAPE	RPIQ
PSO	0.01	10.04	0.65	0.09	0.02	0.88
GWO	0.14	10.04	0.30	0.06	0.00	1.07
CSO	0.62	10.11	1.81	0.06	0.02	0.14
MFO	0.04	10.16	1.13	0.06	0.02	1.50
FPO	0.49	10.11	0.48	0.08	0.01	0.59
BRO	0.68	0.29	1.88	0.03	0.02	1.57

**Table 12:** Soil available phosphorus with different optimization techniques for medium levels.

	$R^{2}$	RMSE	RPD	RRMSE	MAPE	RPIQ
PSO	0.05	11.01	1.04	0.07	0.08	1.32
GWO	0.00	10.86	0.22	0.14	0.03	1.33
CSO	0.06	11.55	1.11	0.17	0.02	1.31
MFO	0.05	11.03	1.13	0.11	0.08	0.63
FPO	0.07	10.76	1.18	0.16	0.10	1.25
BRO	0.15	1.94	1.09	0.12	0.11	1.42

**Table 13:** Soil available EC with different optimization techniques.

	$R^{2}$	RMSE	RPD	RRMSE	MAPE	RPIQ
PSO	0.29	10.27	0.55	0.18	0.63	0.39
GWO	0.06	10.28	0.84	0.88	0.33	0.11
CSO	0.03	10.16	1.10	0.90	0.76	0.35
MFO	0.30	10.12	1.02	0.18	0.58	0.22
FPO	0.28	10.27	0.15	0.70	0.43	0.40
BRO	0.32	0.34	1.22	0.88	1.15	0.48

**Table 14:** Soil OC with different optimization techniques for full levels.

	$R^{2}$	RMSE	RPD	RRMSE	MAPE	RPIQ
PSO	0.06	10.11	0.91	0.19	0.19	0.42
GWO	0.06	10.11	1.11	0.19	0.03	0.90
CSO	0.01	10.12	0.22	0.27	0.17	0.35
MFO	0.00	10.06	0.78	0.08	0.10	0.99
FPO	0.09	10.03	0.43	0.17	0.17	0.28
BRO	0.12	0.14	1.07	0.35	0.21	1.59

**Table 15:** Soil OC with different optimization techniques for low levels.

	$R^{2}$	RMSE	RPD	RRMSE	MAPE	RPIQ
PSO	0.35	10.03	0.74	0.08	0.02	1.20
GWO	0.11	10.01	0.35	0.09	0.07	1.65
CSO	0.29	10.03	1.16	0.11	0.09	1.34
MFO	0.33	10.01	0.39	0.10	0.04	1.39
FPO	0.25	10.03	1.03	0.06	0.03	1.59
BRO	0.43	0.04	1.35	0.09	0.08	2.05

**Table 16:** Soil OC with different optimization techniques for high levels.

	$R^{2}$	RMSE	RPD	RRMSE	MAPE	RPIQ
PSO	0.07	10.04	0.56	0.11	0.05	1.09
GWO	0.18	10.01	0.94	0.06	0.05	0.92
CSO	0.05	10.01	1.28	0.07	0.05	0.19
MFO	0.37	10.03	1.43	0.10	0.05	0.58
FPO	0.08	10.02	0.21	0.10	0.03	1.14
BRO	0.48	0.04	1.44	0.05	0.04	2.08

**Table 17:** Soil pH with different optimization techniques for full levels.

	$R^{2}$	RMSE	RPD	RRMSE	MAPE	RPIQ
PSO	0.09	10.29	0.74	0.13	0.01	1.08
GWO	0.02	10.00	0.69	0.06	0.05	0.91
CSO	0.05	10.23	0.47	0.07	0.03	0.52
MFO	0.11	10.50	0.70	0.10	0.01	0.37
FPO	0.06	10.11	1.02	0.11	0.03	0.47
BRO	0.13	0.62	1.08	0.09	0.07	1.79

**Table 18:** Soil pH with different optimization techniques for high levels.

	$R^{2}$	RMSE	RPD	RRMSE	MAPE	RPIQ
PSO	0.18	10.10	1.16	0.08	0.01	0.23
GWO	0.19	10.02	0.70	0.07	0.01	1.01
CSO	0.10	10.11	0.20	0.07	0.01	0.11
MFO	0.14	10.04	0.28	0.06	0.01	0.28
FPO	0.35	10.06	1.04	0.06	0.01	1.56
BRO	0.45	0.17	1.36	0.02	0.02	2.02

**Table 19:** Soil pH with different optimization techniques for low levels.

	$R^{2}$	RMSE	RPD	RRMSE	MAPE	RPIQ
PSO	0.17	10.07	1.55	0.06	0.01	0.20
GWO	0.26	10.04	0.45	0.06	0.01	1.12
CSO	0.32	10.09	0.79	0.06	0.01	0.68
MFO	0.15	10.10	1.47	0.08	0.00	0.71
FPO	0.13	10.05	1.29	0.07	0.01	0.90
BRO	0.52	0.15	1.48	0.02	0.02	1.91

**Table 20:** Soil pH with different optimization techniques for medium levels.

	$R^{2}$	RMSE	RPD	RRMSE	MAPE	RPIQ
PSO	0.02	10.15	0.90	0.09	0.03	1.58
GWO	0.17	10.17	0.60	0.06	0.01	1.73
CSO	0.01	10.12	1.19	0.10	0.02	0.06
MFO	0.08	10.17	0.70	0.10	0.01	0.83
FPO	0.16	10.25	0.63	0.10	0.02	1.91
BRO	0.35	0.29	1.16	0.04	0.03	1.89

## Data Availability

The raw data supporting the conclusions of this article will be made available by the authors on request.
